# TLR7/TLR9- and B Cell Receptor-Signaling Crosstalk: Promotion of Potentially Dangerous B Cells

**DOI:** 10.3389/fimmu.2017.00775

**Published:** 2017-07-13

**Authors:** Amy N. Suthers, Stefanie Sarantopoulos

**Affiliations:** ^1^Department of Medicine, Division of Hematological Malignancies and Cellular Therapy, Duke Cancer Institute, Duke University Medical Center, Durham, NC, United States; ^2^Department of Immunology, Duke University Medical Center, Durham, NC, United States

**Keywords:** B cell signaling, TLR9, TLR7, chronic graft-versus-host disease, allogeneic hematopoietic stem cell transplantation, B cell receptor, autoantibody production, B cell biology

## Abstract

B cells are capable of receptor-mediated responses to foreign antigens. Recognition of microbial-derived nucleic acid (NA) by toll-like receptors (TLRs) 7 and 9 in B cells has been substantiated. Endogenous NA released from damaged or dying cells can also be immunogenic in certain contexts and can incite aberrant activation of B cells. When TLR-driven B cell receptor (BCR)-activated B cells are not properly constrained, pathologic autoantibodies are produced. It is also clear that endosomal TLR7/TLR9 can operate in conjunction with BCR. In addition to BCR signaling, a balance between TLR7 and TLR9 is pivotal in the development of B cell autoreactivity. While TLR9 is important in normal memory B cell responses through BCR, TLR9 activation has been implicated in autoantibody production. Paradoxically, TLR9 also plays known protective roles against autoimmunity by directly and indirectly inhibiting TLR7-mediated autoantibody production. Herein, we summarize literature supporting mechanisms underpinning the promotion of pathological BCR-activated B cells by TLR7 and TLR9. We focus on the literature regarding known points of TLR7/TLR9 and BCR crosstalk. Data also suggest that the degree of TLR responsiveness relies on alterations of certain intrinsic B-cell signaling molecules and is also context specific. Because allogeneic hematopoietic stem cell transplantation is a high NA and B cell-activating factor environment, we conclude that B cell studies of synergistic TLR–BCR signaling in human diseases like chronic graft-versus-host disease are warranted. Further understanding of the distinct molecular pathways mediating TLR–BCR synergy will lead to the development of therapeutic strategies in autoimmune disease states.

## Introduction

Toll-like receptor (TLR) responses to nucleic acids (NAs) have been extensively studied in monocytes and macrophages (1). In B cells, TLRs, such as TLR7 and TLR9, have been shown to mediate cell responses to both immunogenic NAs and NA-containing immune complexes (ICs) (2–4). Under normal conditions, B cells can respond immediately to initial microbial insults through NA recognition. B cells can also mount recall responses to previously encountered infectious agents and perpetuate life-long serological memory (5). However, when excessive cellular or tissue damage occurs and B cell responses to endogenous cellular NA are not restrained, autoantibodies and autoimmunity are promoted (6). A body of evidence has elucidated cooperative TLR7 or TLR9 (TLR7/TLR9) and B cell receptor (BCR) activation in aberrantly activated B cells. Further understanding of potential molecular synergy between BCR and TLR7/TLR9 pathways in B cells will enable development of agents that can potentially prevent autoimmune states in patients.

## TLR7 and TLR9 Activation Versus Attenuation of Autoreactive B Cells

A number of murine models have been employed to substantiate the roles of TLR9 and/or TLR7 in the production of DNA-associated and RNA-associated autoantibody production, respectively (Table 1). TLR7-deficient autoimmune-prone mice display reduced or absent RNA-associated antibodies, whereas Tlr9-deficient mice have lower amounts of anti-nucleosome and anti-chromatin antibodies (7–9). A pathogenic role for TLR7 was revealed *via* characterization of the Y chromosome-linked autoimmune accelerating (Yaa) mouse that has known TLR7 overexpression due to gene duplication (10, 11). When Yaa are combined with systemic lupus erythematosus (SLE) mice and the *Tlr9* gene knocked out, mice have increased RNA-associated antibodies, exacerbated clinical symptoms, and accelerated mortality (12). Unexpectedly, in all autoimmune-prone mouse models, including MRL/lpr, B6/lpr, Balb/c-Pristane, B6.Nba2.Yaa, B6 Yaa, and Ali5 deficient in TLR9, RNA-associated antibodies are increased, suggesting a more complex role for TLR9 in SLE (8, 9, 12–17). In fact, on an autoimmune-prone background, *Tlr9* deficiency alone leads to overall increased immune activation, exacerbation of pathogenesis, and in some cases increased mortality (8, 9, 12–15). By contrast, *Tlr7*-deficiency in autoimmune-prone mice leads to a significant decrease in overall immune activation and disease severity (9, 14). Thus, TLR7 and TLR9 have opposing pathogenic and protective roles, respectively, in autoimmune disease.

**Table 1:** TLR7/TLR9 responses have substantiated roles in both autoantibody production and autoimmunity, especially in B cell receptor (BCR)-activated B cells.

	TLR7 and TLR9 functions in B cell autoimmunity	Reference
TLR7	RNA-associated antigen recognition	(11)
	RNA-associated autoantibody production	(9)
	Pathogenic role in development of autoimmunity (murine models) Increased IgG production Increased immune (B and T) cell activation Promoted survival of plasmablast/antibody forming cells Increased systemic lupus erythematosus (SLE)-related mortality and pathogenesis	(7, 14, 18, 20)
TLR9	Endogenous double-stranded deoxyribonucleic acid (dsDNA) and chromatin antigen recognition Anti-dsDNA and chromatin-associated autoantibody production	(8, 9, 14)
	Protective role in autoimmunity (by limiting potentially pathogenic role of TLR7 in murine models) Attenuated IgG production (both total and pathologic) Decreased immune activation Induced of B cell tolerance and cell death Decreased SLE-related mortality and pathogenesis	(12, 13, 15–18, 20)

	**Functional synergy of BCR–TLR7/TLR9 pathways**	

BCR–TLR7/TLR9	BCR activation results in increased TLR9	(29)
	BCR dictates subcellular location of TLR9	(28)
	BCR and TLR7/TLR9 increases proliferation, cytokine, and autoantibody production	(25–27, 29, 32)
	BCR and TLR7 operate together to confer autoimmunity, by attenuating TLR7 tolerance	(35)
	BCR and TLR9 synergize to confer central tolerance	(36)

	**Proximal BCR-signaling components and TLR7/TLR9 autoimmune responses**	

	Syk inhibition of B cells blocked the CpG response	(40)
	Btk and Syk mediate TLR crosstalk	(38, 41, 42)
	Btk is dispensible for TLR7 and 9 (ligands and immune complex) proliferation	(39)
	Lyn negatively regulates: Both anti-RNA and anti-dsDNA antibody production (both global deletion and B-cell specific) IgG class-switching B cell activation Cytokine production (pro-inflammatory) Autoimmune pathology	(44–46)

Nundel et al. found that TLR9 directly constrains BCR–TLR7-dependent responses, suggesting a B-cell intrinsic protective role for TRL9 (18). By contrast, *Tlr7*-deficient B cells are not responsive to DNA-containing ICs and have increased death rates. Interestingly, BCR–TLR9-mediated post-proliferative cell death of B cells when TLR7 is absent can be blocked by the TNF family survival cytokine B cell-activating factor (BAFF). Nickerson et al. observed that TLR9 was associated with anti-dsDNA B cell sequestration and deletion, corroborating a protective role for TLR9 (19). The relative contributions of B cell-intrinsic TLR7 and TLR9 on autoimmunity were addressed by Jackson et al. This group generated mixed bone marrow (BM) chimeras by adoptively transferring BM from wild type, Wiskott–Aldrich syndrome (WAS) protein-deficient, *Was*-deficient-*Tlr7*-deficient, or *Was*-deficient-*Tlr9*-deficient mice with μMT BM (20:80) into lethally irradiated μMT recipient mice (20). In this chimeric WAS model, B cells were the predominant cells rendered WAS-deficient and hyperactive. Since immune dysregulation and autoimmunity was largely confined to the B cell compartment, results suggest that the TLR9 and TLR7 effects were B-cell intrinsic (20). Further studies in B cell-specific knockout models are needed to clarify any impact from the 20% myeloid cells also found in this TLR7/TLR9-deficient chimeric model (20). Together, data highlight a need to better understand the molecular mechanisms that underpin pathological or protective responses of TLR7/TLR9 responses in B cells.

## TLR7– and TLR9–BCR Responses are Limited by Availability and Trafficking of NA Ligand

**Figure 1:**
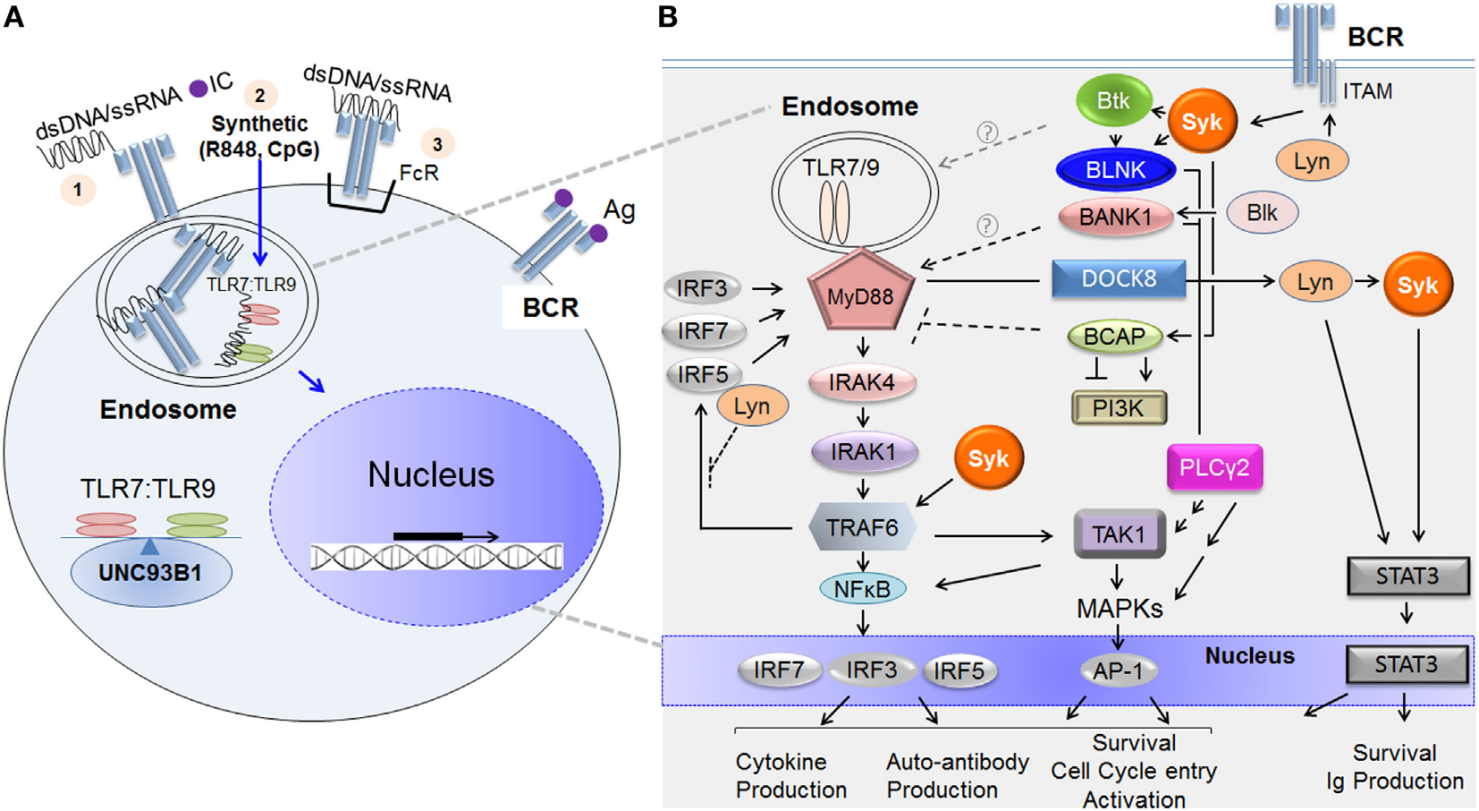
TLR7/TLR9 and B cell receptor (BCR) ligands incite B cell signaling cascades. **(A)** Depiction of how immunogenic extracellular nucleic acid (NA) antigens are internalized to activate endosomal TLR7 and TLR9. NA antigens comprising double-stranded deoxyribonucleic acid (dsDNA) or single-stranded ribonucleic acid (ssRNA) or synthetic analog reach the endosomal compartment *via* any of the following potential mechanisms: (1) membrane uptake of immune complex (IC) (NA protein or antibody); (2) diffusion (synthetic compounds or oligonucleotides), or (3) BCR- or Fc receptor (FcR)-mediated internalization when either is recognized directly. Once internalized, the NA component of the IC binds the appropriate toll-like receptor (TLR) molecule, initiating dimerization and signal transduction. TLR7/TLR9 and BCR pathway stimulation leads to the activation of nuclear factors and to transcription of additional activation genes, such as mediators of proliferation and effector cytokines. Trafficking of TLR7 and TLR9 from the endoplasmic reticulum to endosomal compartments is tightly regulated by the chaperone protein, UNC93B1. **(B)** The major molecular activators immediately downstream of the BCR and TLR7/TLR9 and molecular points of crosstalk between the two signaling pathways. On the left-hand side, initial activation of TLR7/TLR9 by NA in the endosome leads to the recruitment and binding of MyD88 to their intracellular domains. This TLR7/TLR9 activation leads to Myddosome complex composed of MyD88, IRAK1, IRAK4, and subsequent recruitment of TNF receptor-associated factor 6 (TRAF6), each activated sequentially. Ubiquitinated TRAF6 associates with and polyubiquitinates the TAK1 complex (including proteins TAB 1 and TAB 2, not depicted). TAK1 then undergoes autophosphorylation, initiating the MAPK or NFκB pathways. These pathways can each result in the activation of important transcription factors including NFκB, AP-1, and IRFs (3, 5, and 7) that govern B cell fate. On the right-hand side, BCR ligation activates proximal kinase proteins including Lyn, Blk, Syk, and Btk. These kinases phosphorylate adaptor molecules including, BLNK, BCAP, and BANK1, which function as scaffolding proteins and allow for the many divergent pathways activated downstream of BCR including PLCγ2, MAPK, PI3K, and NFκB pathways. Molecules known to convey crosstalk between the BCR–TLR pathways upon ligation by NA-ICs include Lyn, Syk, Btk, BANK1, BCAP, TAK1, and DOCK8. The proposed mechanism of positive or negative regulation of TLR signaling is shown. Key: arrows = activation; multiple arrows = indirect activation; perpendicular lines = inhibition; broken lines = unknown in B cells because published work done on non-B cells; solid lines = protein association; gray broken arrow = contradictory regulation of TLR signaling; circled question-mark = mechanism of TLR regulation unknown. Abbreviations: Lyn, Lck/Yes-related novel protein tyrosine kinase; Blk, B-lymphoid tyrosine kinase; Syk, spleen tyrosine kinase; Btk, Bruton tyrosine kinase; BLNK, B cell linker protein; BCAP, B cell adaptor for phosphoinositide 3-kinase; BANK1, B cell scaffold protein with ankyrin repeats 1; PLCγ2, phosphoinositide-specific C phospholipase gamma 2; PI3K, phosphatidylinositol-4,5-bisphosphate 3-kinase; TAK1, TGFβ-activated kinase 1; MAPK, mitogen-activated kinase; MyD88, myeloid differentiation primary response gene 88; IRAK, interleukin-1 receptor-associated kinase; TRAF, TNF receptor-associated factor; IRF, interferon regulator factor.

TLR7 and TLR9 are located in endosomal compartments and as a consequence, are usually sequestered away from NA-associated ligands. Immunogenic NA is derived from microbes or from damaged or dying cells located in the extracellular matrix (21). In both the physiological and autoimmune settings, endogenous NAs are more likely to form complexes with proteins or antibodies. As depicted in Figure 1A, TLR7/TLR9 ligands like NA-bound proteins can be brought into the B cell *via* several potential mechanisms. Endocytosis of NA-bound protein and diffusion of a synthetic agent (e.g., imiquimod/R848 or CpG) are known examples. Alternatively, NA or NA-ICs can be recognized and internalized by BCRs or Fc receptors and then presented to endosomal TLR7 or TLR9 for subsequent activation (6, 21). Trafficking of TLR7 and TLR9 from the endoplasmic reticulum to endosomal compartments is tightly regulated by the chaperone protein, UNC93B1 (22). The balance of TLR7:TLR9 determines downstream effector function in part because of outcompetition of TLR9 binding to UNC93B1 (23, 24).

Dual engagement of BCR and activation of TLR7/TLR9 were first shown in seminal papers by Marshak-Rothsteins’ group (25, 26). These investigators employed transgenic (Tg) mice that express rheumatoid factor (RF) AM14 BCR. AM14 BCR specifically binds with low affinity to IgG2a that is bound to endogenous or synthetic, highly purified NA. These IgG-NA ICs are “dual specific” and bind to BCR and various forms of NA (chromatin, dsNDA, RNA, SnRNP). A series of studies using this unique set of tools has now substantiated a requirement for BCR-IC internalization in TLR7/TLR9-mediated autoantibody production (25–27).

## TLR7/TLR9 Activation of B Cells Relies on BCR Activation in Certain Contexts

The role of the BCR is not simply to internalize and present NA antigen. After BCR activation, both total and endosomal TLR9 levels increase, suggesting that BCR directly regulates TLR9 (28, 29) (Table 1). Several signaling molecules downstream of BCR operate in concert with TLR pathways to modulate TLR responses (30, 31). In the healthy state, dual BCR and TLR7/TLR9 engagement confer synergistic responses, including cytokine production, antibody production, and class-switch recombination (32, 33). In autoimmune disease, synergistic BCR–TLR7/TLR9 activation by NA-IC results in increased B cell proliferation and autoantibody production (25–27). For full activation of autoreactive RF-B cells, combined signals from the BCR and either TLR7/TLR9 are required (30, 34). Dual engagement of BCR and TLR9 by chromatin-IC leads to distinct functional outcomes (29). BCR activation can operate with TLR7 to attenuate peripheral B cell tolerance (35). Conversely, BCR–TLR9 synergy induces central tolerance through AID expression in autoreactive immature/T1 B cells (36). Together, data reveal a pivotal role for BCR in regulating TLR7 and TLR9 responses for maintaining the balance between normal and pathological B cell activation. A unique pattern of gene expression induced after co-stimulation with BCR and chromatin-ICs versus stimulation with TLR9 or BCR alone further suggests synergistic BCR–TLR signaling (29) Specific molecular mechanisms underpinning BCR–TLR signaling crosstalk are emerging as summarized below.

## Proximal BCR-Signaling Components Important for TLR7/TLR9 Autoimmune Responses

Data reveal that BCR-proximal kinases Syk, Btk, and Lyn are involved in BCR–TLR7/TLR9 crosstalk (Table 1). The proximal BCR-signalosome molecules, Syk and Btk, have been associated with TLR7/TLR9 activation (37–39). In human B cells, Syk appears to be a positive regulator of TLR and TLR–BCR synergism, since inhibition of Syk blocks TLR responses (40, 41). The role of Btk in BCR–TLR signaling is less clear. Btk has been implicated in downstream signaling of TLRs, including TLR9 in B cells (42). Studies in the autoimmune setting revealed that Btk was dispensable for TLR7/TLR9 or BCR–TLR IC responses. In the absence of *Btk*, AM14-Tg B cells had diminished responses to BCR–TLR IC that was related to increased overall cell death, rather than to the level of activation of individual B cells (39). Lyn, a src kinase molecule associated with the positive and negative regulation of the BCR pathway (43) has been shown to negatively regulate TLR7/TLR9 activation (44–46). *Lyn*-deficient or B cell-specific *Lyn*-deficient mouse models had increased NA-associated autoantibodies, cytokine production (including IL-6 and IL-10), and autoimmune pathology (44–46). The exact mechanism of this negative regulatory role in TLR signaling has not been defined in B cells, although it is well established that Lyn is required to phosphorylate and activate CD22, an important negative regulator of BCR signaling (43). While the mechanistic role for Btk remains somewhat contradictory, roles for Lyn and Syk are more defined.

## BCR and TLR7/TLR9 Signaling Cascades: Crosstalk and Potential Aberrant B Cell Activation

Figure 1B is a simplified depiction of the major molecular components TLR7/TLR9 and BCR signaling. As depicted on the left in Figure 1B, TLR7 and TLR9, unlike BCR, signal in a MyD88-dependent fashion. After ligand associates with TLR7 or TLR9 in the endosomal compartment, TLR monomers dimerize and recruit the adaptor protein MyD88 to the intracellular domains. MyD88 then binds the kinase interleukin-1 receptor-associated kinase (IRAK)-4, promoting its autophosphorylation. IRAK4 subsequently associates with and phosphorylates IRAK1 (47). The resultant multimeric MyD88–IRAK4–IRAK1 complex (often referred to as the “Myddosome”) is critical for downstream effector signaling. Phosphorylation of IRAKs is required for recruitment of the E3 ubiquitin ligase TNF receptor-associated factor 6 (TRAF6) to the complex (48). TRAF6, together with two other ubiquitin-conjugating enzymes (not depicted), itself becomes ubiquitinated before translocation into the cytosol where it activates transforming growth factor-beta-activated kinase 1 (TAK1) (48). TAK1 activation results in the phosphorylation and subsequent activation of MAPKs and/or NFκB. NFκB activation leads to phosphorylation of downstream transcription factors, including interferon regulatory factors (IRFs)—IRF3, IRF5, or IRF7. After phosphorylation by TRAF6, these IRFs are translocated to the nucleus where they ultimately determine cell fate (47, 49, 50). As shown on the right in Figure 1B, BCR activation incites signaling through activation of proximal BCR molecules Syk, Lyn, and Btk. When soluble antigen ligates the BCR or when BCR is cross-linked by anti-IgM surrogate antigen, several src kinases including Lyn are rapidly activated. In turn, immuno-receptor tyrosine-based activation motifs (ITAM) within the cytoplasmic domains of the CD79a/CD79b heterodimer complex of the BCR are phosphorylated by Lyn (43). Dual phosphorylation of ITAM tyrosine residues allows the association and subsequent activation of Syk tyrosine kinase (51). Syk can then associate with and activate a number of other kinases, including Btk and adaptor proteins such as B cell linker protein (BLNK) and B cell adaptor for phosphoinositide 3-kinase (BCAP) (51). Another adaptor protein, B cell scaffold protein with ankyrin repeats 1 (BANK1) is primarily activated by B-lymphoid tyrosine kinase (Blk) (52). Adaptor proteins, BANK1 and BCAP, lack kinase activity but function as scaffold proteins in the formation of macromolecular complexes that enable efficient effector signal transduction. Subsequent downstream signals include PLCγ2 activation, calcium mobilization, MAPK, NFκB pathways, and BCAP–PI3K-mediated pathways (52–54).

Improved understanding of distinct molecular mediators of BCR–TLR crosstalk in normal versus aberrant B-cell signaling is emerging. As shown in Figure 1B, BCR-proximal kinases Lyn and Syk have been specifically implicated in molecular BCR–TLR7/TLR9 crosstalk. Lyn has been shown to negatively regulate TLR activation *in vivo* (44–46). While not yet studied directly in B cells, the molecular mechanism may be similar to that found in dendritic cells, where Lyn directly associates with IRF5 and in doing so, inhibits the ability of TRAF6 to associate with and activate the transcription factor (55). Syk is a positive regulator of TLR signaling (40, 41). Syk activation has been associated with TRAF6 expression in B cells from patients with SLE (38). The association between Syk and TRAF6 suggests an important point of crosstalk in the context of autoimmune disease, suggesting a potential mechanism for how Syk blockade attenuates the TLR9 responses (40).

B cell adaptor proteins, BCAP and BANK1, are also potential components of BCR-endosomal TLR signaling crosstalk (Figure 1B). BCAP negatively regulates inflammatory responses mediated by TLRs 4, 7, and 9 by linking TLR–PI3K pathways (56–58). This has been shown to occur through a hidden TIR domain in the full-length BCAP protein, which allows its direct association with TLR adaptor proteins (58, 59). Recently, Halabi et al. published that BCAP binds directly with TLR adaptor proteins to facilitate PLCγ2- and PI3K-mediated depletion of the cell membrane phospholipid component of macrophages (59). Without these phospholipid substrates, the TLR adaptor proteins could not associate with the cell membrane. This potentially results in inhibition of subsequent signal transduction (59). Similar mechanisms downstream of endosomal TLR7/TLR9 may be utilized by B cells, but this requires further investigation. B cell adaptor proteins may also positively regulate TLR7/TLR9. BANK1 in B cells has been shown to augment TLR7/TLR9 signaling; however, the exact mechanism remains unknown (60, 61). Splenic B cells from *Bank1*-deficient mice had significantly decreased TLR9 responses mediated *via* a p38MAPK-dependent mechanism (61). This positive regulatory role of BANK1 was further supported in the autoimmune setting. *Bank1*-deficient mice crossed with B6Sle1.Yaa mice (the TLR7 overexpressing autoimmune model) resulted in reduced IgG production (importantly the pathogenic-associated IgG2a isotype) and reduced IL-6 and BAFF serum levels (60). This was confirmed to be B-cell intrinsic, as *ex vivo* TLR7 responses were impaired (60). Thus, early evidence has identified important roles for BCR adaptor proteins in aberrant B cell responses to TLR in autoimmune disease.

Two other proteins have been implicated in BCR–TLR crosstalk. TAK1 has been suggested to be at the exact point of synergism between the BCR–TLR9 pathways (62). Synergistic BCR–TLR9 activation was abrogated when TAK1 was inhibited and silenced (62). Jabara et al. identified the adaptor dedicator of cytokinesis-8 (DOCK8) as the link between TLR9 and STAT3 activation in B cells (63). This link was dependent upon the BCR-signaling components Lyn and Syk (63). CpG activation of TLR9 induced migration of the preexisting complex consisting of MyD88, DOCK8, and Pyk2 to the cytoplasmic tails of TLR9. This association induced phosphorylation of DOCK8 by Pyk2 and allowed Lyn to bind to DOCK8. Binding of DOCK8 to Lyn leads to subsequent activation of Syk, and activation of STAT3, resulting in gene expression important for long-lived memory B cell survival and antibody production (63). Importantly, DOCK8 was not required for initial BCR signaling. This suggests a pivotal role for the integration of DOCK8 and TLR–BCR signaling cascades in BCR activation by low affinity antigen, although studies examining simultaneous BCR and TLR9 activation are required to address this. Thus, in the physiological context NA-ICs when low affinity BCR is activated before NA is presented to TLR9, DOCK8, Syk, and Lyn may all play even more significant roles in integration of BCR–TLR9 signaling.

## Future Studies: TLR–BCR-Mediated Autoimmunity in Humans

We now know there are a number of molecular links between BCR and TLR7/TLR9 signaling. Further studies are required to determine distinct pathologic signaling pathways so that agents can be used to block aberrant TLR/BCR signaling in patients. Future studies should address mechanisms that restrain potentially dangerous responses to antigen in autoimmune-provoking environments. To do this, more physiological research tools are required to further define mechanisms of TLR–BCR signaling, particularly for studies of human B cells. Many studies use anti-IgM surrogate antigen for the BCR-activation component, which does not precisely recapitulate the more physiological setting of BCR–TLR activation by ICs that requires internalization. Additional technical challenges need to be addressed. Potentially pathogenic B cells are already in an activated state, hampering the ability to stimulate and delineate meaningful mechanistic studies *ex vivo* without inducing cell death. Gene knockdown is also challenging in primary disease-state B cells. Until these technical and logistical barriers are overcome, studies of synergistic BCR–TLR signal transduction in the physiological setting in human B cells remain challenging.

Understanding B-cell intrinsic BCR–TLR signaling and activation in the context of human disease will also require investigation of extrinsic factors involved in the promotion of autoreactive B cells. BAFF plays a pivotal role in B cell development and the maintenance of B cell homeostasis (64). Elevated BAFF levels have been implicated in breaking B cell tolerance in systemic autoimmune diseases including SLE and Sjorgren’s syndrome (SS) (65). Elevated serum BAFF levels have been correlated with circulating autoantibodies, disease progression, and anti-dsDNA antibodies in SLE patients (66, 67) and with autoantibody levels in SS patients (68). One mechanism by which BAFF breaks this tolerance in a lupus-like disease model has been shown to be TLR-dependent (69). Data suggest a model whereby excess BAFF expands autoreactive B cells, and BAFF signals directly promote TLR activation and internalization of dsDNA or NA-IC autoreactive BCRs. BAFF also increases TLR7/TLR9 expression, and TLR7/TLR9 signaling promotes BAFF receptor expression, thus providing a positive feedback loop (69). Further study will elucidate how the extrinsic factor BAFF dysregulates intrinsic BCR–TLR B cell signals and promotes aberrant B cell activation and pathogenesis.

## Implications for a Disease that Develops in an Autoimmune-Provoking Environment: Allogeneic Hematopoietic Stem Cell Transplantation (HCT)

Current evidence as summarized above compels examination of aberrant B cells in patients with autoimmune pathology. This includes patients who develop chronic graft-versus-host disease (cGVHD) that develops after HCT. cGVHD is a B-cell mediated autoimmune disease-like state that is unacceptably debilitating and difficult to treat. Persistently altered B cell homeostasis in patients with cGVHD is potentially perpetuated by global B cell depletion strategies (70–72). In cGVHD, intrinsic abnormalities in the proximal BCR machinery of B cells are being defined. Thus, we and others are interested in developing ways to target only B cells from patients with cGVHD that are hyperactivated and primed for survival *in vivo via* BAFF- and BCR-associated pathways (73). Based on murine and human studies that demonstrated a role for BCR-activated B cells in the pathophysiology of cGVHD (74, 75), the novel application of signaling pathway inhibitors is being tested in clinical trials.

After HCT, B cells are recovering in an NA and alloantigen (76) rich environment that may promote pathological B cells. Circulating monocytes in cGVHD patients upregulate gene pathways involved in innate cellular damage responses (77). Some of these genes include TLR7, BAFF and Type 1 interferons. No definitive examination of TLR7/TLR9 in cGVHD has yet been performed, but studies suggest that there is a muted signaling response to TLR9 agonists by plasmablast-like cells that normally regulate immune responses *via* the production of cytokines including IL-10 (78). We conclude that studies of cGVHD addressing TLR9 and TLR7 signaling of BCR-activated B cells after HCT are warranted. Such studies will inevitably lead to further understanding of human B cell tolerance and will likely compel the expanded use of targeted therapeutic agents in patients.

## Author Contributions

SS and AS both researched the topic, wrote and edited the manuscript, and made the table and figure for this manuscript.

## Conflict of Interest Statement

The authors declare that the research was conducted in the absence of any commercial or financial relationships that could be construed as a potential conflict of interest.

## References

[1] Kawasaki T Kawai T. Toll-like receptor signaling pathways. Front Immunol (2014) 5:461. 10.3389/fimmu.2014.00461 25309543 PMC4174766

[2] Green NM Marshak-Rothstein A. Toll-like receptor driven B cell activation in the induction of systemic autoimmunity. Semin Immunol (2011) 23(2):106–12. 10.1016/j.smim.2011.01.016 21306913 PMC3070769

[3] Marshak-Rothstein A. Toll-like receptors in systemic autoimmune disease. Nat Rev Immunol (2006) 6(11):823–35. 10.1038/nri1957 17063184 PMC7097510

[4] Jimenez-Dalmaroni MJ Gerswhin ME Adamopoulos IE The critical role of toll-like receptors – from microbial recognition to autoimmunity: a comprehensive review. Autoimmun Rev (2016) 15(1):1–8. 10.1016/j.autrev.2015.08.009 26299984 PMC4679489

[5] Kurosaki T Kometani K Ise W. Memory B cells. Nat Rev Immunol (2015) 15(3):149–59. 10.1038/nri3802 25677494

[6] Rifkin IR Leadbetter EA Busconi L Viglianti G Marshak-Rothstein A Toll-like receptors, endogenous ligands, and systemic autoimmune disease. Immunol Rev (2005) 204(1):27–42. 10.1111/j.0105-2896.2005.00239.x 15790348

[7] Berland R Fernandez L Kari E Han JH Lomakin I Akira S Toll-like receptor 7-dependent loss of B cell tolerance in pathogenic autoantibody knockin mice. Immunity (2006) 25(3):429–40. 10.1016/j.immuni.2006.07.014 16973388

[8] Christensen SR Kashgarian M Alexopoulou L Flavell RA Akira S Shlomchik MJ. Toll-like receptor 9 controls anti-DNA autoantibody production in murine lupus. J Exp Med (2005) 202(2):321–31. 10.1084/jem.20050338 16027240 PMC2212997

[9] Christensen SR Shupe J Nickerson K Kashgarian M Flavell RA Shlomchik MJ. Toll-like receptor 7 and TLR9 dictate autoantibody specificity and have opposing inflammatory and regulatory roles in a murine model of lupus. Immunity (2006) 25(3):417–28. 10.1016/j.immuni.2006.07.013 16973389

[10] Fairhurst A-M Hwang S-h Wang A Tian X-H Boudreaux C Zhou XJ Yaa-autoimmune phenotypes are conferred by an overexpression of TLR7. Eur J Immunol (2008) 38(7):1971–8. 10.1002/eji.200838138 18521959 PMC2993003

[11] Pisitkun P Deane JA Difilippantonio MJ Tarasenko T Satterthwaite AB Bolland S. Autoreactive B cell responses to RNA-related antigens due to TLR7 gene duplication. Science (2006) 312(5780):1669–72. 10.1126/science.1124978 16709748

[12] Santiago-Raber M-L Dunand-Sauthier I Wu T Li Q-Z Uematsu S Akira S Critical role of TLR7 in the acceleration of systemic lupus erythematosus in TLR9-deficient mice. J Autoimmun (2010) 34(4):339–48. 10.1016/j.jaut.2009.11.001 19944565

[13] Lartigue A Courville P Auquit I Francois A Arnoult C Tron F Role of TLR9 in anti-nucleosome and anti-DNA antibody production in lpr mutation-induced murine lupus. J Immunol (2006) 177(2):1349–54. 10.4049/jimmunol.177.2.1349 16818796

[14] Nickerson KM Christensen SR Shupe J Kashgarian M Kim D Elkon K TLR9 regulates TLR7- and MyD88-dependent autoantibody production and disease in a murine model of lupus. J Immunol (2010) 184(4):1840–8. 10.4049/jimmunol.0902592 20089701 PMC4098568

[15] Bossaller L Christ A Pelka K Nundel K Chiang PI Pang C TLR9 deficiency leads to accelerated renal disease and myeloid lineage abnormalities in pristane-induced murine lupus. J Immunol (2016) 197(4):1044–53. 10.4049/jimmunol.1501943 27354219 PMC5266544

[16] Yu P Wellmann U Kunder S Quintanilla-Martinez L Jennen L Dear N Toll-like receptor 9-independent aggravation of glomerulonephritis in a novel model of SLE. Int Immunol (2006) 18(8):1211–9. 10.1093/intimm/dxl067 16798839

[17] Wu X Peng SL. Toll-like receptor 9 signaling protects against murine lupus. Arthritis Rheum (2006) 54(1):336–42. 10.1002/art.21553 16385525

[18] Nundel K Green NM Shaffer AL Moody KL Busto P Eilat D Cell-intrinsic expression of TLR9 in autoreactive B cells constrains BCR/TLR7-dependent responses. J Immunol (2015) 194(6):2504–12. 10.4049/jimmunol.1402425 25681333 PMC4382804

[19] Nickerson KM Christensen SR Cullen JL Meng W Luning Prak ET Shlomchik MJ. TLR9 promotes tolerance by restricting survival of anergic anti-DNA B cells, yet is also required for their activation. J Immunol (2013) 190(4):1447–56. 10.4049/jimmunol.1202115 23296704 PMC3563726

[20] Jackson SW Scharping NE Kolhatkar NS Khim S Schwartz MA Li Q-Z Opposing impact of B cell-intrinsic TLR7 and TLR9 signals on autoantibody repertoire and systemic inflammation. J Immunol (2014) 192(10):4525–32. 10.4049/jimmunol.1400098 24711620 PMC4041708

[21] Pelka K Shibata T Miyake K Latz E. Nucleic acid-sensing TLRs and autoimmunity: novel insights from structural and cell biology. Immunol Rev (2016) 269(1):60–75. 10.1111/imr.12375 26683145

[22] Kim YM Brinkmann MM Paquet ME Ploegh HL. UNC93B1 delivers nucleotide-sensing toll-like receptors to endolysosomes. Nature (2008) 452(7184):234–8. 10.1038/nature06726 18305481

[23] Fukui R Saitoh S Kanno A Onji M Shibata T Ito A Unc93B1 restricts systemic lethal inflammation by orchestrating toll-like receptor 7 and 9 trafficking. Immunity (2011) 35(1):69–81. 10.1016/j.immuni.2011.05.010 21683627

[24] Fukui R Saitoh S Matsumoto F Kozuka-Hata H Oyama M Tabeta K Unc93B1 biases toll-like receptor responses to nucleic acid in dendritic cells toward DNA- but against RNA-sensing. J Exp Med (2009) 206(6):1339–50. 10.1084/jem.20082316 19451267 PMC2715051

[25] Lau CM Broughton C Tabor AS Akira S Flavell RA Mamula MJ RNA-associated autoantigens activate B cells by combined B cell antigen receptor/toll-like receptor 7 engagement. J Exp Med (2005) 202(9):1171–7. 10.1084/jem.20050630 16260486 PMC2213226

[26] Leadbetter EA Rifkin IR Hohlbaum AM Beaudette BC Shlomchik MJ Marshak-Rothstein A. Chromatin-IgG complexes activate B cells by dual engagement of IgM and toll-like receptors. Nature (2002) 416(6881):603–7. 10.1038/416603a 11948342

[27] Viglianti GA Lau CM Hanley TM Miko BA Shlomchik MJ Marshak-Rothstein A. Activation of autoreactive B cells by CpG dsDNA. Immunity (2003) 19(6):837–47. 10.1016/S1074-7613(03)00323-6 14670301

[28] Chaturvedi A Dorward D Pierce SK. The B cell receptor governs the subcellular location of toll-like receptor 9 leading to hyperresponses to DNA-containing antigens. Immunity (2008) 28(6):799–809. 10.1016/j.immuni.2008.03.019 18513998 PMC2601674

[29] Busconi L Bauer JW Tumang JR Laws A Perkins-Mesires K Tabor AS Functional outcome of B cell activation by chromatin immune complex engagement of the B cell receptor and TLR9. J Immunol (2007) 179(11):7397–405. 10.4049/jimmunol.179.11.7397 18025183

[30] Rawlings DJ Schwartz MA Jackson SW Meyer-Bahlburg A. Integration of B cell responses through toll-like receptors and antigen receptors. Nat Rev Immunol (2012) 12(4):282–94. 10.1038/nri3190 22421786 PMC3437941

[31] Rawlings DJ Metzler G Wray-Dutra M Jackson SW Altered B cell signalling in autoimmunity. Nat Rev Immunol (2017). 10.1038/nri.2017.24 PMC5523822 28393923

[32] Pone EJ Zhang J Mai T White CA Li G Sakakura JK BCR-signalling synergizes with TLR-signalling for induction of AID and immunoglobulin class-switching through the non-canonical NF-kappaB pathway. Nat Commun (2012) 3:767 10.1038/ncomms1769 22473011 PMC3337981

[33] Vanden Bush TJ Bishop GA. TLR7 and CD40 cooperate in IL-6 production via enhanced JNK and AP-1 activation. Eur J Immunol (2008) 38(2):400–9. 10.1002/eji.200737602 18228247 PMC2951126

[34] Avalos AM Busconi L Marshak-Rothstein A. Regulation of autoreactive B cell responses to endogenous TLR ligands. Autoimmunity (2010) 43(1):76–83. 10.3109/08916930903374618 20014959 PMC3059585

[35] Poovassery JS Vanden Bush TJ Bishop GA. Antigen receptor signals rescue B cells from TLR tolerance. J Immunol (2009) 183(5):2974–83. 10.4049/jimmunol.0900495 19648281 PMC2789010

[36] Kuraoka M Snowden PB Nojima T Verkoczy L Haynes BF Kitamura D BCR and endosomal TLR signals synergize to increase AID expression and establish central B cell tolerance. Cell Rep (2017) 18(7):1627–35. 10.1016/j.celrep.2017.01.050 28199836 PMC5328188

[37] Iwata S Tanaka Y. B-cell subsets, signaling and their roles in secretion of autoantibodies. Lupus (2016) 25(8):850–6. 10.1177/0961203316643172 27252261

[38] Iwata S Yamaoka K Niiro H Jabbarzadeh-Tabrizi S Wang S-P Kondo M Increased Syk phosphorylation leads to overexpression of TRAF6 in peripheral B cells of patients with systemic lupus erythematosus. Lupus (2015) 24(7):695–704. 10.1177/0961203314560424 25432781

[39] Nundel K Busto P Debatis M Marshak-Rothstein A. The role of Bruton’s tyrosine kinase in the development and BCR/TLR-dependent activation of AM14 rheumatoid factor B cells. J Leukoc Biol (2013) 94(5):865–75. 10.1189/jlb.0313126 23804807 PMC3800065

[40] Kremlitzka M Mácsik-Valent B Erdei A. Syk is indispensable for CpG-induced activation and differentiation of human B cells. Cell Mol Life Sci (2015) 72(11):2223–36. 10.1007/s00018-014-1806-x 25543269 PMC11113211

[41] Iwata S Yamaoka K Niiro H Nakano K Wang SP Akashi K Amplification of toll-like receptor-mediated signaling through spleen tyrosine kinase in human B-cell activation. J Allergy Clin Immunol (2012) 129(6):1594–601.e2. 10.1016/j.jaci.2012.03.014 22541243

[42] Lee KG Xu S Wong ET Tergaonkar V Lam KP. Bruton’s tyrosine kinase separately regulates NFkappaB p65RelA activation and cytokine interleukin (IL)-10/IL-12 production in TLR9-stimulated B cells. J Biol Chem (2008) 283(17):11189–98. 10.1074/jbc.M708516200 18276597

[43] Xu Y Harder KW Huntington ND Hibbs ML Tarlinton DM Lyn tyrosine kinase: attenuating the positive and the negative. Immunity (2005) 22(1):9–18. 10.1016/j.immuni.2004.12.004 15664155

[44] Hua Z Gross AJ Lamagna C Ramos-Hernández N Scapini P Ji M Requirement for MyD88 signaling in B cells and dendritic cells for germinal center anti-nuclear antibody production in Lyn-deficient mice. J Immunol (2014) 192(3):875–85. 10.4049/jimmunol.1300683 24379120 PMC4101002

[45] Lamagna C Hu Y DeFranco AL Lowell CA. B cell-specific loss of Lyn kinase leads to autoimmunity. J Immunol (2014) 192(3):919–28. 10.4049/jimmunol.1301979 24376269 PMC3900234

[46] Nishizumi H Taniuchi I Yamanashi Y Kitamura D Ilic D Mori S Impaired proliferation of peripheral B cells and indication of autoimmune disease in Lyn-deficient mice. Immunity (1995) 3(5):549–60. 10.1016/1074-7613(95)90126-4 7584145

[47] De Nardo D. Toll-like receptors: activation, signalling and transcriptional modulation. Cytokine (2015) 74(2):181–9. 10.1016/j.cyto.2015.02.025 25846205

[48] Qian Y Commane M Ninomiya-Tsuji J Matsumoto K Li X IRAK-mediated translocation of TRAF6 and TAB 2 in the interleukin-1-induced activation of NFκB. J Biol Chem (2001) 276(45):41661–7. 10.1074/jbc.M102262200 11518704

[49] Sato S Sanjo H Takeda K Ninomiya-Tsuji J Yamamoto M Kawai T Essential function for the kinase TAK1 in innate and adaptive immune responses. Nat Immunol (2005) 6(11):1087–95. 10.1038/ni1255 16186825

[50] Honda K Taniguchi T. IRFs: master regulators of signalling by toll-like receptors and cytosolic pattern-recognition receptors. Nat Rev Immunol (2006) 6(9):644–58. 10.1038/nri1900 16932750

[51] Geahlen RL. Syk and pTyr’d: signaling through the B cell antigen receptor. Biochim Biophys Acta (2009) 1793(7):1115–27. 10.1016/j.bbamcr.2009.03.004 19306898 PMC2700185

[52] Bernal-Quirós M Wu Y-Y Alarcón-Riquelme ME Castillejo-López C. BANK1 and BLK act through phospholipase C gamma 2 in B-cell signaling. PLoS One (2013) 8(3):e59842. 10.1371/journal.pone.0059842 23555801 PMC3608554

[53] Kurosaki T Tsukada S BLNK: connecting Syk and Btk to calcium signals. Immunity (2000) 12(1):1–5. 10.1016/S1074-7613(00)80153-3 10661400

[54] Okada T Maeda A Iwamatsu A Gotoh K Kurosaki T. BCAP: the tyrosine kinase substrate that connects B cell receptor to phosphoinositide 3-kinase activation. Immunity (2000) 13(6):817–27. 10.1016/S1074-7613(00)00079-0 11163197

[55] Ban T Sato GR Nishiyama A Akiyama A Takasuna M Umehara M Lyn kinase suppresses the transcriptional activity of IRF5 in the TLR-MyD88 pathway to restrain the development of autoimmunity. Immunity (2016) 45(2):319–32. 10.1016/j.immuni.2016.07.015 27521268

[56] Matsumura T Oyama M Kozuka-Hata H Ishikawa K Inoue T Muta T Identification of BCAP-L as a negative regulator of the TLR signaling-induced production of IL-6 and IL-10 in macrophages by tyrosine phosphoproteomics. Biochem Biophys Res Commun (2010) 400(2):265–70. 10.1016/j.bbrc.2010.08.055 20728433

[57] Ni M MacFarlane AW Toft M Lowell CA Campbell KS Hamerman JA B-cell adaptor for PI3K (BCAP) negatively regulates toll-like receptor signaling through activation of PI3K. Proc Natl Acad Sci U S A (2012) 109(1):267–72. 10.1073/pnas.1111957108 22187458 PMC3252908

[58] Troutman TD Hu W Fulenchek S Yamazaki T Kurosaki T Bazan JF Role for B-cell adapter for PI3K (BCAP) as a signaling adapter linking toll-like receptors (TLRs) to serine/threonine kinases PI3K/Akt. Proc Natl Acad Sci U S A (2012) 109(1):273–8. 10.1073/pnas.1118579109 22187460 PMC3252926

[59] Halabi S Sekine E Verstak B Gay NJ Moncrieffe MC. Structure of the toll/interleukin-1 receptor (TIR) domain of the B-cell adaptor that links phosphoinositide metabolism with the negative regulation of the toll-like receptor (TLR) signalosome. J Biol Chem (2017) 292(2):652–60. 10.1074/jbc.M116.761528 27909057 PMC5241739

[60] Wu YY Kumar R Iida R Bagavant H Alarcon-Riquelme ME. BANK1 regulates IgG production in a lupus model by controlling TLR7-dependent STAT1 activation. PLoS One (2016) 11(5):e0156302. 10.1371/journal.pone.0156302 27228057 PMC4882053

[61] Wu Y-Y Kumar R Haque MS Castillejo-López C Alarcón-Riquelme ME. BANK1 controls CpG-induced IL-6 secretion via a p38 and MNK1/2/eIF4E translation initiation pathway. J Immunol (2013) 191(12):6110–6. 10.4049/jimmunol.1301203 24227780 PMC3858538

[62] Szili D Banko Z Toth EA Nagy G Rojkovich B Gati T TGFbeta activated kinase 1 (TAK1) at the crossroad of B cell receptor and toll-like receptor 9 signaling pathways in human B cells. PLoS One (2014) 9(5):e96381 10.1371/journal.pone.0096381 24801688 PMC4011794

[63] Jabara HH McDonald DR Janssen E Massaad MJ Ramesh N Borzutzky A DOCK8 functions as an adaptor that links TLR-MyD88 signaling to B cell activation. Nat Immunol (2012) 13(6):612–20. 10.1038/ni.2305 22581261 PMC3362684

[64] Mackay F Browning JL. BAFF: a fundamental survival factor for B cells. Nat Rev Immunol (2002) 2(7):465–75. 10.1038/nri844 12094221

[65] Nakayamada S Tanaka Y BAFF- and APRIL-targeted therapy in systemic autoimmune diseases. Inflamm Regen (2016) 36(1):6 10.1186/s41232-016-0015-4 PMC5725651 29259679

[66] Chu VT Enghard P Schurer S Steinhauser G Rudolph B Riemekasten G Systemic activation of the immune system induces aberrant BAFF and APRIL expression in B cells in patients with systemic lupus erythematosus. Arthritis Rheum (2009) 60:2083–93. 10.1002/art.24628 19565488

[67] Zollars E Bienkowska J Czerkowicz J Allaire N Ranger AM Magder L BAFF (B cell activating factor) transcript level in peripheral blood of patients with SLE is associated with same-day disease activity as well as global activity over the next year. Lupus Sci Med (2015) 2:e000063. 10.1136/lupus-2014-000063 26113988 PMC4477150

[68] Mariette X Roux S Zhang J Bengoufa D Lavie F Zhou T The level of BLyS (BAFF) correlates with the titre of autoantibodies in human Sjögren’s syndrome. Ann Rheum Dis (2003) 62(2):168–71. 10.1136/ard.62.2.168 12525388 PMC1754442

[69] Groom JR Fletcher CA Walters SN Grey ST Watt SV Sweet MJ BAFF and MyD88 signals promote a lupuslike disease independent of T cells. J Exp Med (2007) 204(8):1959–71. 10.1084/jem.20062567 17664289 PMC2118661

[70] Sarantopoulos S Ritz J. Aberrant B-cell homeostasis in chronic GVHD. Blood (2015) 125(11):1703–7. 10.1182/blood-2014-12-567834 25645355 PMC4357579

[71] Sarantopoulos S Stevenson KE Kim HT Cutler CS Bhuiya NS Schowalter M Altered B-cell homeostasis and excess BAFF in human chronic graft-versus-host disease. Blood (2009) 113(16):3865–74. 10.1182/blood-2008-09-177840 19168788 PMC2670799

[72] Sarantopoulos S Stevenson KE Kim HT Washel WS Bhuiya NS Cutler CS Recovery of B-cell homeostasis after rituximab in chronic graft-versus-host disease. Blood (2011) 117(7):2275–83. 10.1182/blood-2010-10-307819 21097674 PMC3062333

[73] Allen JL Fore MS Wooten J Roehrs PA Bhuiya NS Hoffert T B cells from patients with chronic GVHD are activated and primed for survival via BAFF-mediated pathways. Blood (2012) 120(12):2529–36. 10.1182/blood-2012-06-438911 22896003 PMC3448264

[74] Allen JL Tata PV Fore MS Wooten J Rudra S Deal AM Increased BCR responsiveness in B cells from patients with chronic GVHD. Blood (2014) 123(13):2108–15. 10.1182/blood-2013-10-533562 24532806 PMC3968393

[75] Flynn R Allen JL Luznik L MacDonald KP Paz K Alexander KA Targeting Syk-activated B cells in murine and human chronic graft-versus-host disease. Blood (2015) 125(26):4085–94. 10.1182/blood-2014-08-595470 25852057 PMC4481596

[76] Ramadan A Paczesny S. Various forms of tissue damage and danger signals following hematopoietic stem-cell transplantation. Front Immunol (2015) 6:14. 10.3389/fimmu.2015.00014 25674088 PMC4309199

[77] Hakim FT Memon S Jin P Imanguli MM Wang H Rehman N Upregulation of IFN-inducible and damage-response pathways in chronic graft-versus-host disease. J Immunol (2016) 197(9):3490–503. 10.4049/jimmunol.1601054 27694491 PMC5101132

[78] de Masson A Bouaziz JD Le Buanec H Robin M O’Meara A Parquet N CD24(hi)CD27(+) and plasmablast-like regulatory B cells in human chronic graft-versus-host disease. Blood (2015) 125(11):1830–9. 10.1182/blood-2014-09-599159 25605369

